# Serum metabolomic profiling of human gastric cancer and its relationship with the prognosis

**DOI:** 10.18632/oncotarget.21314

**Published:** 2017-09-28

**Authors:** Daguang Wang, Wei Li, Qi Zou, Lei Yin, Yechao Du, Jingkai Gu, Jian Suo

**Affiliations:** ^1^ Department of Gastrointestinal Surgery, First Hospital of Jilin University, Changchun, Jilin 130021, China; ^2^ Clinical Pharmacology Center, Research Institute of Translational Medicine, First Hospital of Jilin University, Changchun, Jilin 130021, China

**Keywords:** gastric cancer (GC), metabolomics, serum, biomarkers, prognosis

## Abstract

**Objective:**

This study was aimed to investigate serum metabolites in gastric cancer (GC) patients and their relationships with the prognosis of GC in order to find potential specific serum biomarkers for GC.

**Methods:**

Blood samples of 125 GC patients of unifocal GC at initial stage and 38 healthy people recruited in our hospital from September 2008 to August 2009 were analyzed by using high performance liquid chromatography coupled with electrospray ionization/quadrupole-time-of-flight mass spectrometry (HPLCESI/Q-TOFMS). Multiple statistical methods like principal component analysis (PCA), hierarchical clustering analysis, partial least squares discriminant analysis (PLS-DA), multivariate COX regression analysis, variance analysis and K-M survival curve were applied to analyze the raw obtained mass data in order to analyze the independent prognostic factors of GC. The structures of these metabolites were confirmed by comparing the m/z ratio and ion mode of with the data published from HMDB (www.hmdb.ca) databases.

**Results:**

By PLS-DA test, 16 serum metabolites in ESI^+^ mode of VIP>1 in both test group and validation group could definitely distinguish GC patients from healthy peoples (*p*<0.05). Multivariate COX regression analysis showed TNM staging, 2,4-hexadienoic acid, 4-methylphenyl dodecanoate and glycerol tributanoate were independent prognostic factors of GC (*p*<0.05). In the K-M survival analysis, the survival rate in high level group of the 3 selected serum metabolites together or alone was significant lower than in those in low level group (*p*<0.05).

**Conclusion:**

Low serum levels of 2,4-hexadienoic acid, 4-methylphenyl dodecanoate and glycerol tributanoate may be important independent prognostic factors of GC.

## INTRODUCTION

Developed from the malignant cells in the stomach inner lining, gastric cancer (GC) has high mortality all over the world, commonly in the Eastern countries, such as in China, Korea and Japan [1–3]. Nowadays, therapies for GC consist of chemotherapy, surgery, radiation and targeted therapy [4–6]. As the underlying molecular mechanism of GC is still unknown and the clinical symptoms of early gastric cancer are usually unobvious, there still lacks of effective therapy for GC.

Endoscopy is the most common diagnostic method for the early GC, but the efficiency was inconsistent among different endoscopists and pathologists [6, 7]. Although early definition and management at the beginning stage can decrease GC incidence, the prognosis of GC remains poor and the overall 5-year survival rate is still less than 40% [4–6]. The current prognosis indicators include pathology findings of histological type, invasion and metastasis, imaging results of classifications and other clinical characteristics like age and underlying diseases, etc., and further therapy method should be determined based on all these indicators. However, there still lots of limitations for these traditional indicators. Currently, biomarkers like p27, cyclin E, E-cadherin, c-erbB2, cmyc, tumor suppressor gene p53 etc. have been reported to be effective prognosis factors for GC patients. Furthermore, more and more serum metabolomics have been recommended as prognosis indicators in developed countries due to high specificity and sensitivity, which plays a key role in GC therapy [8, 9].

Lots of biological studies show that metabolites in human fluid samples (such as serum, bile, sputum, aqueous humor, etc.) can be important downstream or endpoint biomarkers for gene mutation mutations due to endogenous substance or xenobiotics and are more specific and sensitive to different disease stages [10–14]. As a result, metabolomics plays an important role in current biological research, especially in underlying carcinogenesis and proliferation mechanism and thereby establishing useful biomedical indicators for early diagnosis and management in current cancer research, such as breast cancer, prostate cancer, lung cancer, colorectal cancer, pancreatic esophageal cancer, ovarian cancer, bladder cancer, renal cancer, etc. [10–14]. With the application of modern chromatography and mass spectrometry or other detection techniques like nuclear magnetic resonance, lots of biological metabolites have proven by numbers of statistical methods (such as t-test, discriminant analysis, principal component analysis, cluster analysis etc.) to be specific and sensitive biomarkers in current cancer research [10–14]. Recently, some clinical studies showed that biological metabolites in the fluid or tissue samples were of great beneficial in the early diagnosis and managements for GC [15–19]. However, there were still few studies investigated on the serum metabolites might be novel diagnostic indicators for gastrointestinal cancer [20–23].

In this study, we aimed to analyze serum metabolites between GC patients and the healthy people as well as their relationship with the prognosis of GC in order to find potential specific and sensitive serum biomarkers for GC by using high performance liquid chromatography coupled with electrospray ionization/quadrupole-time-of-flight mass spectrometry (HPLCESI/Q-TOFMS), which can be of clinical beneficial for the early diagnosis and management of GC.

## RESULTS

### Total ion current spectras between GC patients and healthy people in serum samples

**Figure 1:**
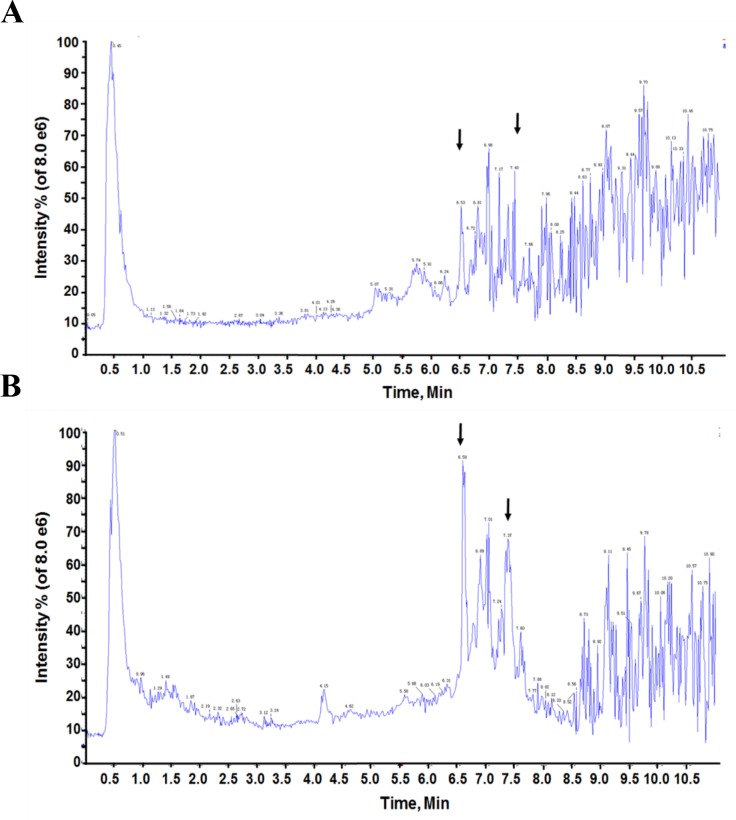
A representative serum total ion current mass spectra of GC patients and healthy people **(A)** serum sample of GC patient, **(B)** serum sample of healthy control. The arrows indicated two markedly different wave crests between A and B.

In this study, all the biological metabolites in serum samples from both GC patients and healthy people were detected by using HPLCESI/Q-TOFMS, providing lots of information for clarifying unknown molecular mechanisms for GC. There were obvious difference in the total ion current spectras of serum samples between GC patients and healthy people under the full-scan mode (in retention time of 11 minutes), which suggested that there might be some important metabolic changes in GC patients (Figure 1). By using compared t- test, a total of 87 metabolites in ESI^+^ mode were find to be statistically different between GC patients and healthy people (*p*<0.05) (Table 1). However, there were no statistically different serum metabolites in ESI^-^ mode between GC patients and healthy people (Figure not shown). The clear identifications of each biological metabolites and their related effect in the biological processes are critical for the metabolomics’ researches. Compared with the data obtained from HMDB (www.hmdb.ca) databases, 87 metabolites in ESI^+^ mode were structurally confirmed.

**Table 1:** Biological metabolites identified from serum samples between GC patients and healthy controls by LC-MS/MS

No.	m/z ratio	Name	Response in GC patients	Response in healthy controls
1	104.1051	3-Aminobutanoic acid	73.19 ±20.12	5.60 ±1.34
2	112.8931	2,4-2,4-hexadienoic acid	56.19 ±10.83	4.66 ±1.03
3	224.0947	Benzoic acid	400.33 ±65.14	31.63 ±9.38
4	246.242	Valerylcarnitine	121.88 ±41.71	34.26 ±23.65
5	267.6473	Tetradecylcyclobutanone	5737.45 ±1396.97	1973.43 ±249.91
6	274.2739	Heptanoylcarnitine	8842.87 ±1862.07	297.34 ±63.54
7	279.6473	Alpha-Linolenic acid	3367.18 ±687.08	199.02 ±59.96
8	280.2627	Octanamide	51.51 ±17.31	7.03 ±7.03
9	280.655	NA^a^	1328.04 ±277.34	28.86 ±9.89
10	282.2789	Petroselinic acid	575.40 ±240.94	137.69 ±46.56
11	290.2688	hydroxyhexanoyl carnitine	106.32 ±22.24	27.37 ±25.74
12	291.6468	Etiocholanolone	507.15 ±133.22	16.10 ±9.62
13	301.1413	4-Methoxybenzyl glucoside	180.99 ±43.56	13.19 ±3.02
14	302.3057	Nonanoylcarnitine	4585.55 ±1731.78	499.35 ±189.16
15	303.2282	Glycerol tributanoate	354.91 ±57.12	18.69 ±7.33
16	304.2609	7-Hydroxyetodolac	275.46 ±87.01	56.67 ±26.29
17	318.3004	Phytosphingosine	8913.46 ±2027.48	400.52 ±94.27
18	325.2106	Cibaric acid	327.67 ±91.65	23.10 ±5.29
19	330.3373	Dihydroceramide	6873.61 ±3931.61	196.00 ±58.97
20	346.3319	3-hydroxyundecanoyl carnitine	1371.12 ±402.15	6.53 ±6.53
21	356.2026	S-Adenosylmethioninamine	211.11 ±84.51	10.12 ±3.49
22	358.3678	Cyclodopa glucoside	3736.15 ±2409.30	24.56 ±6.27
23	362.3263	Eicosatrienoic acid isobutylamide	2214.32 ±508.10	28.30 ±28.30
24	374.2451	3-hydroxytridecanoyl carnitine	174.38 ±69.82	5.15 ±1.06
25	374.3624	Pipereicosalidine	1102.80 ±223.45	296.22 ±116.53
26	374.3638	Benzenediol	1872.03 ±670.08	37.08 ±10.08
27	381.1301	3-O-Methylglycyrol	421.97 ±81.57	128.83 ±59.19
28	385.2925	Persicachrome	609.10 ±145.62	42.08 ±10.25
29	390.358	NA^a^	593.58 ±104.37	127.06 ±35.64
30	397.1042	Aloesol 7-glucoside	596.67 ±107.17	64.38 ±16.04
31	398.2422	PGF2a ethanolamide	180.03 ±63.98	5.27 ±1.02
32	399.3074	methyltetracosanoic acid	594.49 ±186.36	23.85 ±20.64
33	402.395	3-hydroxypentadecanoyl carnitine	2103.19 ±624.11	34.71 ±12.66
34	413.2659	Abscisic alcohol 11-glucoside	638.29 ±260.28	42.65 ±10.10
35	415.2098	4-O-Methylmelleolide	1673.09 ±159.29	680.56 ±180.15
36	418.3871	(E)-Casimiroedine	107.79 ±31.43	5.13 ±1.04
37	429.319	Sorbitan oleate	2048.02 ±392.91	66.25 ±19.67
38	437.1937	Phenethyl 6-galloylglucoside	2428.94 ±626.37	1416.72 ±296.65
39	443.334	4-carboxylic acid	585.14 ±152.89	47.68 ±11.64
40	453.1674	Melleolide M	3263.71 ±829.68	65.07 ±36.92
41	457.3499	3-Hydroxycycloart-24-en-21-oic acid	76.99 ±26.06	4.46 ±1.00
42	463.3028	Lucidenic acid M	127.09 ±48.34	18.37 ±14.39
43	473.3451	20beta-Hydroxyursolic acid	1415.31 ±283.51	61.94 ±11.68
44	495.1719	3’-O-beta-glucuronide	546.95 ±114.95	84.87 ±84.87
45	508.2495	2-Decarboxybetanin	120.77 ±39.00	34.96 ±8.12
46	523.2461	Physangulide	378.89 ±96.94	47.19 ±9.89
47	545.26	Cinncassiol A 19-glucoside	701.86 ±144.20	74.31 ±13.06
48	567.2735	Hordatine A	1346.25 ±237.43	208.17 ±50.16
49	568.4302	Neoacrimarine H	1889.75 ±284.80	275.31 ±48.47
50	574.2894	Biotinyl-5’-AMP	390.73 ±167.63	24.03 ±24.03
51	580.2921	Pelargonidin 3-rhamnoside 5-glucoside	4141.02 ±987.75	27.99 ±20.16
52	585.2696	Bilirubin	734.33 ±204.06	150.44 ±68.99
53	589.2874	D-Urobilin	1928.48 ±327.89	182.80 ±35.41
54	590.4269	NA^a^	758.58 ±209.97	50.96 ±13.75
55	601.2987	Gluten exorphin	780.67 ±190.11	36.48 ±8.94
56	604.2921	Neocasomorphin	1302.89 ±255.42	81.58 ±24.79
57	606.3082	Amphibine H	1014.17 ±188.93	125.36 ±17.64
58	611.3	Endomorphin	2765.37 ±432.30	104.87 ±67.11
59	633.3133	Coagulin R 3-glucoside	4175.29 ±640.38	138.07 ±123.68
60	637.4743	9-Oxoasimicinone	1147.96 ±314.53	156.41 ±32.71
61	638.2497	Heparan sulfate	666.21 ±132.75	84.87 ±19.96
62	648.2786	Indoleacetyl glutamine	729.88 ±239.73	28.15 ±6.35
63	648.3096	Nummularine A	1444.89 ±267.22	42.69 ±8.35
64	659.2882	Gluten exorphin	9057.13 ±1693.18	120.98 ±38.72
65	663.3058	Physalolactone B 3-glucoside	1128.26 ±309.07	44.65 ±15.35
66	669.4128	Isolimonic acid glucoside	537.72 ±173.15	26.31 ±20.60
67	670.3232	Nummularine B	2960.59 ±526.46	62.81 ±7.90
68	677.3397	Cucurbitacin I 2-glucoside	7285.01 ±1103.93	270.65 ±53.18
69	681.8775	Omphalotin A	505.93 ±151.78	28.23 ±6.85
70	685.3196	Neoacrimarine B	1139.76 ±344.13	29.83 ±8.00
71	692.3353	Alpha-Tetrasaccharide	4325.35 ±671.19	231.51 ±54.84
72	699.3534	Corchorusoside A	7137.84 ±1140.89	169.75 ±63.62
73	700.2265	Pteroyltriglutamic acid	550.30 ±163.99	19.08 ±16.40
74	710.2494	Fasciculic acid C	745.48 ±204.58	21.68 ±21.68
75	714.3493	Delphinidin 3-5-glucoside	4938.62 ±839.57	85.83 ±37.91
76	718.4179	Glutamate	282.12 ±133.46	24.85 ±19.14
77	721.3659	PG	8637.88 ±1248.68	185.68 ±34.10
78	743.3773	Hordatine B glucoside	7222.37 ±1169.08	160.23 ±29.39
79	751.3585	28-Glucosyloleanolic acid	2299.14 ±501.59	378.22 ±96.16
80	758.3772	3-sambubioside 5-glucoside	6708.47 ±1087.93	410.25 ±85.23
81	765.3931	Momordin I	8359.28 ±1443.52	856.41 ±100.21
82	806.9407	NA^a^	3911.48 ±1009.33	412.08 ±85.32
83	836.5099	Sulfogalactosylceramide	4608.88 ±792.23	210.96 ±118.04
84	853.443	Hovenidulcioside A1	2695.84 ±650.38	282.23 ±85.97
85	875.4587	Pectenotoxin	1639.73 ±515.89	69.48 ±10.31
86	907.3786	Isohopeaphenol	473.66 ±271.60	17.46 ±3.42
87	912.4675	(3S)-3-Hydroxyadipyl-CoA	1326.56 ±333.41	76.58 ±8.54

**Note:** Values of response were expressed as mean ± SD. NA^a^: not available.

**Table 2:** Biological metabolites identified from serum samples of GC patients by LC-MS/MS

No.	m/z ratio	Name	Response	VIP value
M1	279.6473	Alpha-Linolenic acid	3367.18 ±687.08	1.077265
M2	381.1301	3-O-Methylglycyrol	421.97 ±81.57	0.976792
M3	397.1042	Aloesol 7-glucoside	596.67 ±107.17	1.114385
M4	415.2098	4-O-Methylmelleolide	1673.09 ±159.29	1.09627
M5	523.2461	Physangulide	378.89 ±96.94	0.933432
M6	545.26	Cinncassiol A 19-glucoside	701.86 ±144.20	0.956085
M7	567.2735	Hordatine A	1346.25 ±237.43	1.001599
M8	574.2894	Biotinyl-5’-AMP	390.73 ±167.63	1.120802
M9	585.2696	Bilirubin	734.33 ±204.06	0.964372
M10	589.2874	D-urobilin	1928.48 ±327.89	0.964866
M11	590.4269	NA^a^	758.58 ±209.97	0.936668
M12	601.2987	Gluten exorphin	780.67 ±190.11	0.926127
M13	604.2921	Neocasomorphin	1302.89 ±255.42	1.059444
M14	606.3082	Amphibine H	1014.17 ±188.93	0.93589
M15	611.3	Endomorphin	2765.37 ±432.30	1.03234
M16	633.3133	Coagulin R 3-glucoside	4175.29 ±640.38	1.020049
M17	637.4743	9-Oxoasimicinone	1147.96 ±314.53	0.924135
M18	648.2786	Indoleacetyl glutamine	729.88 ±239.73	0.814445
M19	659.2882	Gluten exorphin	9057.13 ±1693.18	1.057791
M20	663.3058	Physalolactone B 3-glucoside	1128.26 ±309.07	0.96284
M21	669.4128	Isolimonic acid glucoside	537.72 ±173.15	1.039349
M22	670.3232	Nummularine B	2960.59 ±526.46	0.990336
M23	677.3397	Cucurbitacin I 2-glucoside	7285.01 ±1103.93	1.020499
M24	681.8775	Omphalotin A	505.93 ±151.78	0.958896
M25	685.3196	Neoacrimarine B	1139.76 ±344.13	0.906009
M26	692.3353	Alpha-Tetrasaccharide	4325.35 ±671.19	1.024838
M27	699.3534	Corchorusoside A	7137.84 ±1140.89	1.018284
M28	710.2494	Fasciculic acid C	745.48 ±204.58	0.856801
M29	714.3493	Delphinidin 3-5-glucoside	4938.62 ±839.57	0.996077
M30	718.4179	Glutamate	282.12 ±133.46	1.307308
M31	721.3659	PG	8637.88 ±1248.68	1.07165
M32	743.3773	Hordatine B glucoside	7222.37 ±1169.08	1.036024
M33	751.3585	28-Glucosyloleanolic acid	2299.14 ±501.59	0.943374
M34	758.3772	3-sambubioside 5-glucoside	6708.47 ±1087.93	0.997932
M35	765.3931	Momordin I	8359.28 ±1443.52	0.928922
M36	836.5099	Sulfogalactosylceramide	4608.88 ±792.23	0.980611
M37	853.443	Hovenidulcioside A1	2695.84 ±650.38	0.936904
M38	875.4587	Pectenotoxin	1639.73 ±515.89	0.997788
M39	907.3786	Isohopeaphenol	473.66 ±271.60	0.980952

**Note:** Values of response were expressed as mean ± SD. NA^a^: not available. VIP: Variable Importance in Projection

### Principal component analysis (PCA)

**Figure 2:**
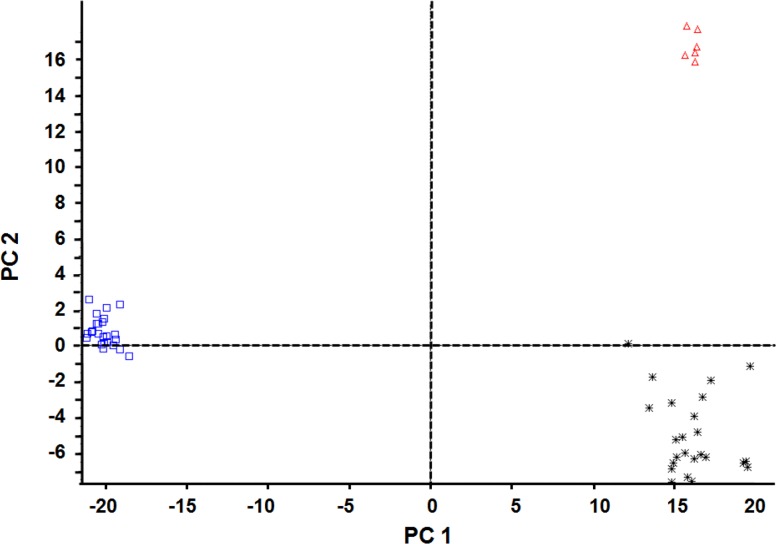
Principal component analysis (PCA) scores plots of serum biological metabolites from GC patients and healthy people in test group in ESI+ mode Blank square (□), healthy people, 24 samples; black star (^*^), GC patients, 24 samples; blank triangle (Δ), quality control, 6 samples. ESI: electrospray ionization.

PCA was adopted as a statistical tool for clustering the detected serum metabolites into smaller number as principal components (PCs) to find the specific metabolic differences between GC patients and healthy controls in test group (24 GC patients and 24 healthy controls) and then to distinguish the outliers or discretization trends in GC patients. In the current study, almost all the samples were clearly grouped or separated in PCA plots, indicating the serum metabolites were properly classified in GC patients and healthy people (Figure 2).

There were obvious difference between GC patients and healthy people under the full-scan mode, but the response in GC patients or healthy people did not vary a lot. By using two sample t-test, a total of 39 metabolites in ESI^+^ mode were found to be statistically different between GC patients and healthy people, which might be of potential power to distinguish the GC patients from the healthy controls (*p*<0.05) (Table 1).

### Partial least squares discriminant analysis (PLS-DA)

**Figure 3:**
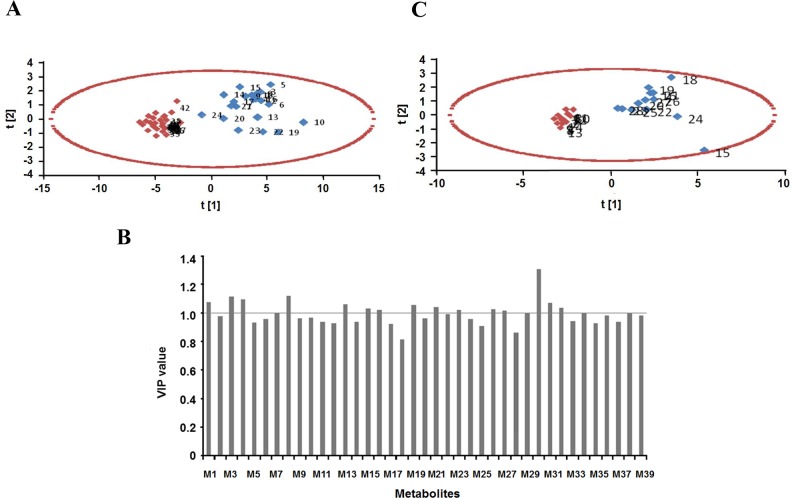
Partial least squares discriminant analysis (PLS-DA) of serum biological metabolites of GC patients and healthy people in ESI+ mode **(A)** PLS-DA score plots in the test group, Red, healthy controls; blue, GC patients; **(B)** VIP plots of PLS-DA analysis in the test group, the line in the figure indicate VIP=1, M1-M39 is the number of 39 metabolites in the Table 2; **(C)** PLS-DA score plots in the validation group. Red, healthy controls; blue, GC patients. ESI: electrospray ionization.

**Figure 4:**
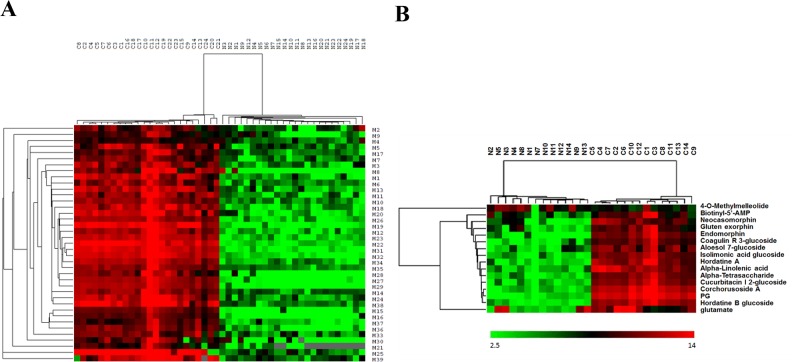
Hierarchical clustering analysis of serum biological metabolites of GC patients and healthy people in ESI^+^ mode in the test group (24 GC patients and 24 healthy controls, 39 serum metabolites determined by PCA) **(A)** and in the validation group (14 GC patients and 14 healthy control, 16 serum metabolites determined by PLS-DA) **(B)**. Red, positive value; green, negative value; black, equal to zero. Each GC patient or healthy people was listed in column. Each serum biological metabolite was listed in row. N: healthy controls; T: GC patients. ESI: electrospray ionization. M1-M39 is the No. of 39 metabolites in the Table 2.

To find the most important serum metabolites between GC patients and healthy controls, partial least squares discriminant analysis (PLS-DA) (Figure 3) was done in test group (24 GC patients and 24 healthy controls). PLS-DA results showed that most of the serum metabolites were clearly clustered in PLS-DA plot with the sensitivity and specificity was 100%. This was consistent with the PCA results, indicating that the 39 serum metabolites could be of statistical importance to separate GC patients and healthy controls. Subsequently, 16 serum metabolites in ESI^+^ mode according to the VIP (Variable Importance in Projection) plots of PLS-DA were found to be highly significant between GC patients and healthy volunteers (VIP>1). Based on above two sample t-tests, a hierarchical clustering analysis on the selected serum metabolites was implemented to visualize the relative significant serum metabolites. Statistically, 24 serum metabolites in ESI^+^ mode in test group could definitely distinguish GC patients from healthy peoples (Figure 4A), furthermore, 16 serum metabolites in ESI^+^ mode of VIP>1 in validation group could definitely distinguish GC patients from healthy peoples (Figure 4B) (*p*<0.05).

### Relationships between serum metabolites with the clinicopathologic features of GC patients

**Figure 5:**
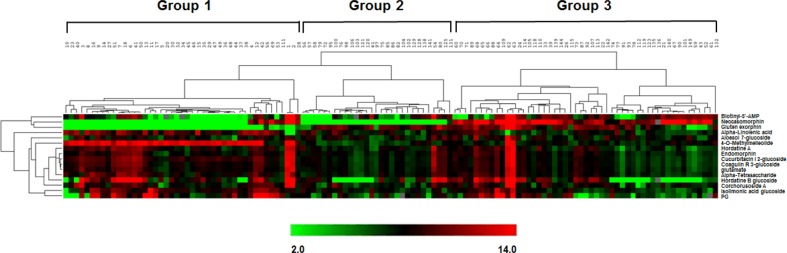
Hierarchical clustering analysis of the 16 selected serum metabolites in ESI+ mode of all the 125 GC patients in this study Red, positive value; green, negative value; black, equal to zero. In the same group of GC patients, responses to the 16 selected serum metabolites were similar. N: healthy controls; T: GC patients. ESI: electrospray ionization.

**Figure 6:**
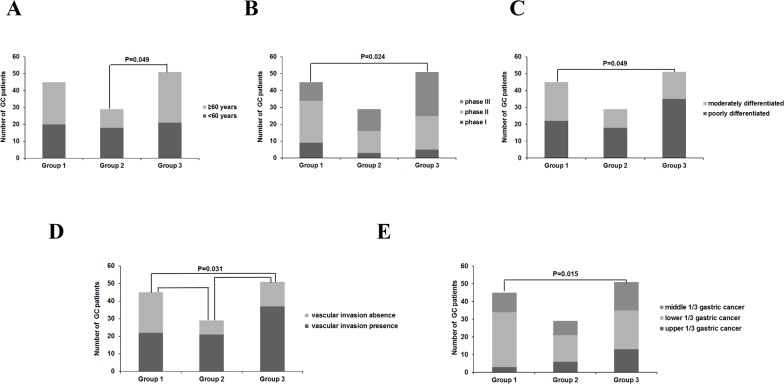
Histograms of different clinicopathologic features of all the 125 GC patients among the 3 groups divided by the 16 selected serum metabolites in ESI+ mode **(A)** Proportion of different age (>60 years and < 60 years), **(B)** Proportion of different TNM staging (phase I, phase II and phase III); **(C)** Proportion of different differentiation type (moderate differentiation and poor differentiation); **(D)** Proportion of different vascular invasion type (presence and absence; **(E)** Proportion of different tumor position (upper gastric cancer, antrum gastric cancer and lower gastric cancer). ESI: electrospray ionization.

**Figure 7:**
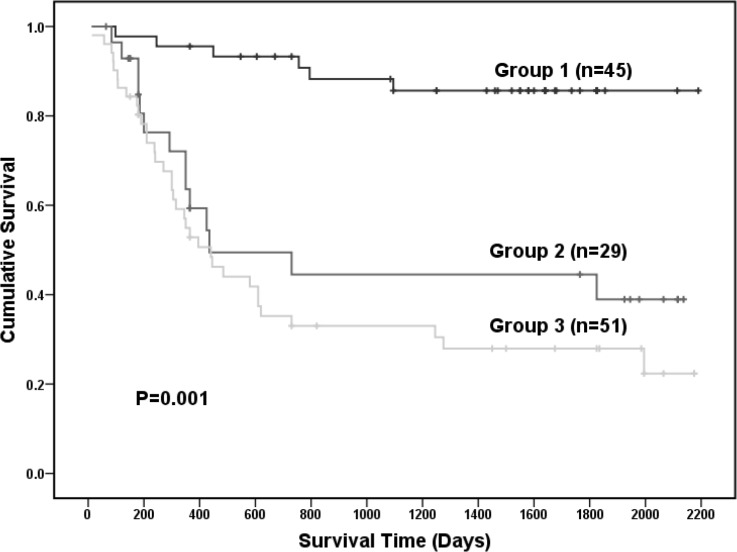
The Kaplan-Meier (K-M) survival curve of all the 125 GC patients among the different groups by the 16 selected serum metabolites in ESI+ mode

According to 16 serum metabolites selected from the PLS-DA results, all the 125 GC patients were divided into 3 groups (Group 1, Group 2 and Group 3) (Figure 5). In the same group of GC patients, responses to the 16 selected serum metabolites were similar. Differences in clinicopathological parameters like tumor differentiation, age, vascular invasion, TNM staging, tumor position and the expression of Ki-67 and P53 were observed among the subgroups by chi-square test (Figure 6). In the GC patients of Group 3, there were significant higher proportion of older patients (>60 years), TNM staging (phase III), poorly differentiated gastric cancer, and upper gastric cancer (*p*<0.05). In the GC patients of Group 1, there were significant higher proportion of vascular invasion and upper gastric cancer (*p*<0.05). By using variance analysis, an increased trend in the expression of Ki-67 and P53 was observed among different groups with the highest level in Group 3 (*p*<0.05). The Kaplan-Meier (K-M) survival curve of each group were plotted and the survival rates were computed correspondingly (Figure 5). Based on the Kaplan-Meier (K-M) survival curve, survival rate statistically vary a lot among the three groups and significant differences in survival time were obviously observed between Group 1 and Groups 2 or Group 3(*p*<0.05) (Figure 7).

### Relationships between serum metabolites with the prognosis of GC

**Figure 8:**
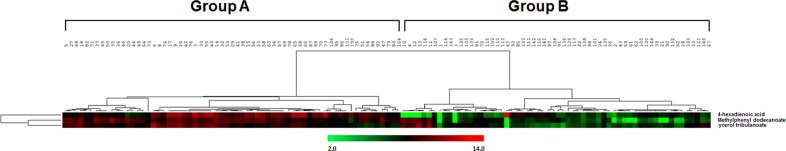
Hierarchical clustering analysis of the 3 selected serum metabolites of all the 125 GC patients in this study **Group A**, high response level; **Group B**, low response level. ESI: electrospray ionization.

**Figure 9:**
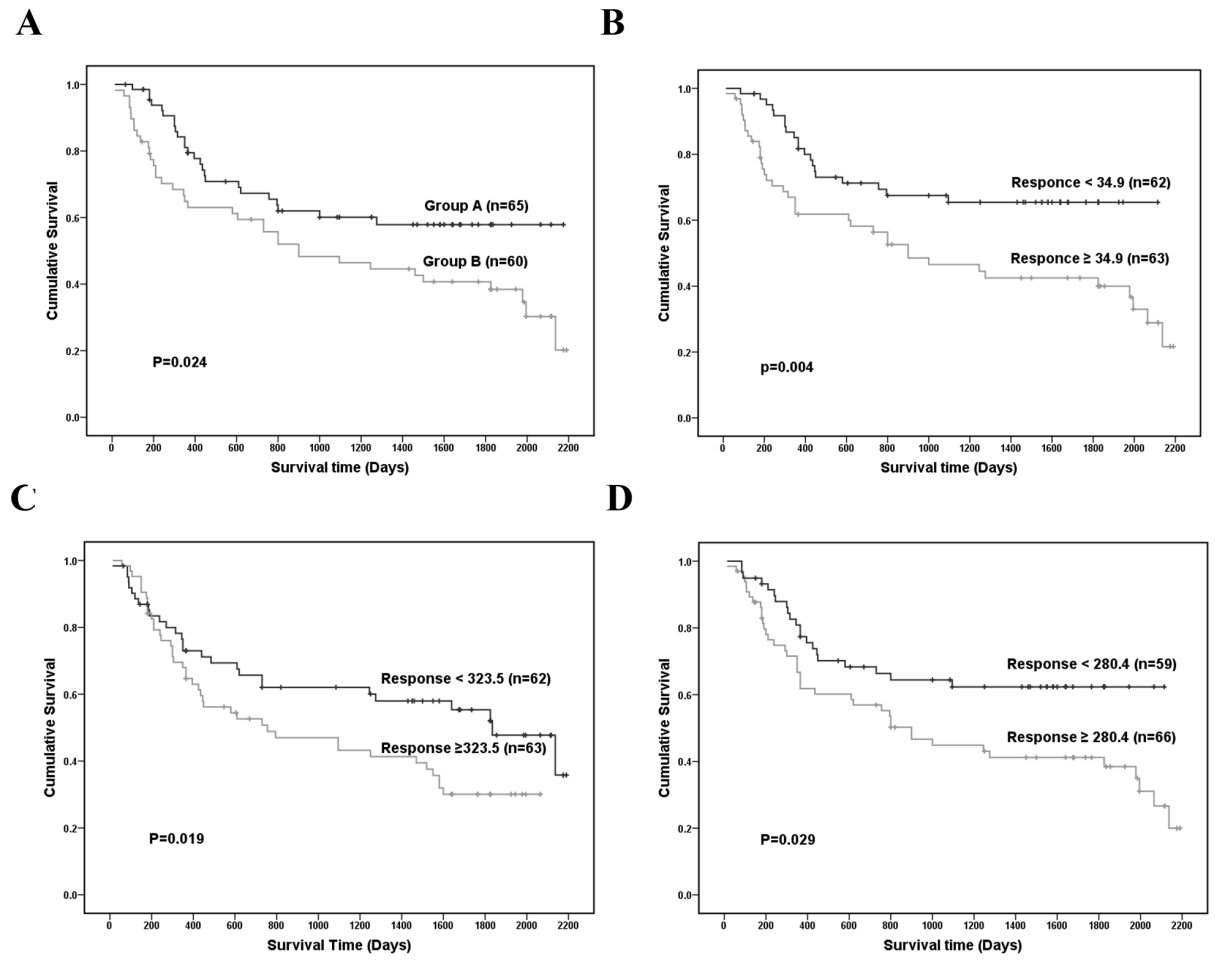
The Kaplan-Meier (K-M) survival curve of all the 125 GC patients among the different groups by the 3 selected serum metabolites in ESI+ mode (2,4-hexadienoic acid, 4-methylphenyl dodecanoate and glycerol tributanoate) **(A)** K-M survival curve of among the different groups by the 3 selected serum metabolites (2,4-hexadienoic acid, 4-methylphenyl dodecanoate and glycerol tributanoate) together; **(B)** K-M survival curve of among the different groups by 2,4-hexadienoic acid only; **(C)** K-M survival curve of among the different groups by 4-methylphenyl dodecanoate only; **(D)** K-M survival curve of among the different groups by glycerol tributanoate only. ESI: electrospray ionization.

As analyzed by univariate analysis of 87 serum metabolites in ESI^+^ mode from all the 125 GC patients in this study, prognostic factors for GC consisted of 2,4-hexadienoic acid, 4-methylphenyl dodecanoate, glycerol tributanoate, methionyl-methionine and PG (*p<0.05*) (Table 3). Subsequently, multivariate COX regression analysis, variance analysis and the K-M survival curve were used to find the independent prognostic factor for GC in human serum. Multivariate COX regression analysis showed TNM staging (*p*<0.005, 95% confidence interval (95% CI) 1.002-1.034), 2,4-hexadienoic acid (*p*<0.005, 95% CI 1.002-1.027), 4-methylphenyl dodecanoate (*p*<0.005, 95% 95% CI 1.002-1.001) and glycerol tributanoate (*p*<0.005, 95% CI 1.002-1.032) were independent prognostic factors of GC (Table 4). In variance analysis, the 3 selected serum metabolites (2,4-hexadienoic acid, 4-methylphenyl dodecanoate and glycerol tributanoate) in ESI^+^ mode in both high level group and low level group could definitely distinguish GC patients from healthy peoples (Figure 8) (*p*<0.05). As shown in the K-M survival curve, the survival rate in high level group of the 3 selected serum metabolites (2,4-hexadienoic acid, 4-methylphenyl dodecanoate and glycerol tributanoate) together was significant lower than in those in low level group of the 3 selected serum metabolites (Figure 9) (*p*<0.05). Consistent with the the K-M survival result with only 1 selected serum metabolite (2,4-hexadienoic acid, 4-methylphenyl dodecanoate or glycerol tributanoate alone) (Figure 9) (*p*<0.05), this might suggest that the 3 selected serum metabolites (2,4-hexadienoic acid, 4-methylphenyl dodecanoate and glycerol tributanoate) can be considered to be useful prognostic factors for GC.

**Table 3:** Univariate analysis of 87 serum metabolites from all the 125 GC patients in this study

Variables	B	SE	P value	OR	95.0% CI for OR	
					Lower	Upper
**2,4-hexadienoic acid**	0.017	0.005	0.002^*^	1.017	1.006	1.027
**4-Methylphenyl dodecanoate**	0.000	0.000	0.013^*^	1.000	1.000	1.001
**Glycerol tributanoate**	0.001	0.000	0.048^*^	1.001	1.000	1.001
**Methionyl-Methionine**	0.002	0.001	0.004^*^	1.002	1.001	1.003
**PG**	0.001	0.000	0.032^*^	1.001	1.000	1.001

**Note:** ^*^p< 0.05 was set to be of statistical significance.

**Table 4:** Multivariate COX regression analysis to find independent prognostic factors of GC

Variables	B	SE	P value	OR	95.0% CI for OR	
					Lower	Upper
**Gender**	0.028	0.307	0.928	1.028	0.563	1.878
**Age**	0.008	0.012	0.493	1.008	0.985	1.031
**Vascular invasion**	0.293	0.287	0.308	1.340	0.763	2.352
**TNM staging**	0.018	0.008	0.024^*^	1.018	1.002	1.034
**Tumor differentiation**	-0.112	0.286	0.696	0.894	0.511	1.565
**2,4-hexadienoic acid**	0.016	0.008	0.025^*^	1.016	1.002	1.027
**4-Methylphenyl dodecanoate**	0.001	0.001	0.001^*^	1.001	1.000	1.001
**Glycerol tributanoate**	0.017	0.008	0.023^*^	1.017	1.002	1.032

**Note:** ^*^p< 0.05 was set to be of statistical significance.

## DISCUSSION

More and more biological studies showed that biological metabolites were “downstream” to genes or “endpoint markers” for disease, and thereby metabolomics is now of global attentions in cancer research in the field of breast cancer, prostate cancer, lung cancer, colorectal cancer, pancreatic esophageal cancer, ovarian cancer, bladder cancer and renal cancer for early diagnosis and effective managements as well as molecular mechanisms. Detected by using modern chromatography and mass spectrometry or other detection techniques, lots of biological metabolites have proven by numbers of statistical methods (such as t-test, partial least squares discriminant analysis, principal component analysis, cluster analysis, etc.) to be potential specific and sensitive biomarkers for different cancer [10–14]. Developed from the malignant cells in the stomach inner lining, gastric cancer (GC) is a major cause of cancer-related death today. Traditional diagnosis methods consist of biopsy, endoscopy and pathological examination [1–3]. However, these diagnosis methods involve with significant limitations, and the efficiency was inconsistent among different endoscopists and pathologists. Recently, some clinical studies showed that biological metabolites in the fluid or tissue samples were of great beneficial in the early diagnosis and managements for GC [15–19]. Whereas, there were still few studies recommended using serum metabolites as a novel diagnostic approach for GC [20–23]. In this study, serum metabolites were investigated between gastric cancer (GC) patients and the healthy people as well as their relationships with the prognosis of GC in order to find potential specific serum biomarkers for GC by using high performance liquid chromatography coupled with electrospray ionization/quadrupole-time-of-flight mass spectrometry (HPLCESI/Q-TOFMS). Statistically, a total of 87 metabolites (Table 1) in ESI+ mode were find to be statistically different between GC patients and healthy people, including 16 serum metabolites in ESI+ mode of VIP>1 in both test group and validation group which could definitely distinguish GC patients from healthy peoples (p<0.05) (Figure 4). According to 16 serum metabolites selected from the PLS-DA results (Table 2), all the 125 GC patients were divided into 3 groups (Group 1, Group 2 and Group 3) (Figure 5). Serum metabolites detected in this study with statistical different responses in between GC patients and healthy people reveal several important metabolic or molecular pathways for GC. Firstly, most gastric cancer cells produce energy primarily through Valsalva effect instead of the citric acid cycle and will change the serum levels of the metabolites of the citric acid cycles [21]. Secondly, the disorder of serum amino acid can influence the cell growth, cell metastasis and cell apoptosis as the raw materials for the protein and nucleic acid synthesis of the cancer cells [24]. Additionally, the disorder of serum fatty acid can also affect the cell growth, cell metastasis and cell apoptosis as well as tumor angiogenesis through untaken or over-exploited by the cancer cell proliferation and growth, or inhibited by the synthesis [9]. Furthermore, other substances like lactate, creatine and succinate were also involved in the metabolic pathways in GC patients comparing with the healthy controls [25].

Clinicopathological parameters like tumor differentiation, age, vascular invasion, TNM staging, survival rate, tumor position and the expression of Ki-67 and P53 were statistically different among the subgroups divided by the 16 serum metabolites selected from the PLS-DA results by the using chi-square test or Kaplan-Meier (K-M) survival curve (p<0.05) (Figure 6). These results was consistent with some prvious studies showing some structural proteins include receptors, membrane channel proteins and enzymes like SRY (sex determining region Y)-box 2, serum gastrin, pepsinogen I and octamer-binding protein-4 (OCT4) plays a vital role in gastric cancer metastasis or differentiation and thereby in TNM staging of GC [26, 27]. Ki-67 and and P53 were highly expressed in GC, and factors influence the expression of Ki-67 and P53 might be important for regulating the cell growth, cell metastasis and cell apoptosis in GC [28].

Currently, serum metabolites like cyclin-dependent kinase 2 (Cdk2), Protein kinase B (Akt) and oleic acid were recommended to be independent prognostic factors of GC [18, 29]. In this metabolomics study, univariate analysis showed that 2,4-hexadienoic acid, 4-methylphenyl dodecanoate, glycerol tributanoate, methionyl-methionine and PG might be the prognostic factors for GC (Table 3). However, TNM staging (*p*<0.005, 95% confidence interval (95% CI) 1.002-1.034), 2,4-hexadienoic acid (*p*<0.005, 95% CI 1.002-1.027), 4-methylphenyl dodecanoate (*p*<0.005, 95% CI 1.002-1.001) and glycerol tributanoate (*p*<0.005, 95% CI 1.002-1.032) were subsequently confirmed to be independent prognostic factors of GC, by using multivariate COX regression analysis (Table 4). Additionally, in the K-M survival analysis, the survival rate in high level group of the 3 selected serum metabolites together or alone was significant lower than in those in low level group (*p*<0.05) (Figure 9). All of the 3 selected serum metabolites (2,4-hexadienoic acid, 4-methylphenyl dodecanoate and glycerol tributanoate) were fatty acid, and high level of the these metabolites in GC patients might be related with the high level of fatty acid synthase regulated by sterol regulatory element-binding proteins (SREBPs) in the fatty acid pathway in GC [30, 31]. All these results might indicate that low serum levels of 2,4-hexadienoic acid, 4-methylphenyl dodecanoate and glycerol tributanoate may be independent prognostic factors of GC.

In the current study, there are some limitations. Firstly, more clinicopathological parameters like gender, weight, BMI, eating habits or other biomarkers for GC like related miRNA or RNA levels like let-7, matrix metalloproteinase levels like MMP-3, MMP7 and MMP-13, COX-2 levels should also be observed. Secondly, the groups should also into more detailed subgroups for each clinicopathological parameters. Finally, other online compound databanks besides HMDB, including METLIN, LIPID MAPS and CEU Mass Mediator, should be used to confirm the chemical structure of the serum metabolites. All these limitations might cause some variation to the results.

To conclude, with the application of modern chromatography and detection techniques as well as different statistical methods, genomics, transcriptomics, proteomics and metabolomics is of key importance in biological studies, especially in the fields of establishing specific potential biomarkers for early diagnosis and effective managements or finding novel molecular mechanisms for the cell growth, cell metastasis and cell apoptosis, tumor angiogenesis in current cancer research, such as breast cancer, prostate cancer, lung cancer, colorectal cancer, pancreatic esophageal cancer, ovarian cancer, bladder cancer, renal cancer, etc. More and more biological studies showed that biological metabolites were highly related with clinicopathological parameters like tumor differentiation, age, vascular invasion, TNM staging, survival rate, tumor position, as well as the prognosis of the cancer. In this metabolomics study, 16 serum metabolites was found to be able to distinguish the GC patients from the healthy controls and 3 serum metabolites (2,4-hexadienoic acid, 4-methylphenyl dodecanoate and glycerol tributanoate) of fatty acid pathways may be independent prognostic factors of GC, which might be of great beneficial for the early diagnosis and management of GC. To conclude, low serum levels of 2,4-hexadienoic acid, 4-methylphenyl dodecanoate and glycerol tributanoate may be important independent prognostic factors of GC.

## MATERIALS AND METHODS

### Study design

Blood samples of 125 GC patients of unifocal GC at initial stage and 38 healthy people recruited in our hospital from September 2008 to August 2009 were analyzed in this study. The blood samples of all the patients and healthy people were extracted in the morning and the basal metabolic rate (BMR) were in the normal range. All the patients were divided into 3 groups: the test group (24 GC patients and 24 healthy controls), the validation group (14 GC patients and 14 healthy control) and the additional group (87 GC patients). There were no significantly different in the basic clinicopathological factors, such as age, sex, BMI, etc between GC patients and healthy people in both the test and the validation group. Both the test and the validation group were investigated to compare the differences serum metabolites between GC patients and healthy controls in order to find the potential specific biomarkers for GC. Besides, blood samples of all the 125 patients were analyzed to find the relationship between biological metabolites and clinical parameters of GC and find specific prognostic factors for GC.

All the included GC patients had a complete 5-year follow-up record and did not have any hormone therapy or chemotherapy before, and all of them were with no significant acute inflammatory disease, normal liver and kidney function, routine physical status, normal results of biochemical tests and electrocardiograp (ECG). The patients should not have congenital disease for the last 2 weeks like burns, severe trauma, and septic shock, metabolic diseases like diabetes, severe heart and lung, liver or kidney disease, neurological and psychiatric diseases, blood diseases like leukemia and anemia, chronic inflammatory diseases, infectious diseases like HIV, hepatitis, and active tuberculosis, or any acute illnesses or stress reactions, etc. The lactation and pregnancy or possible pregnancy women, drinker, drug addicts, long-term user of proton pump inhibitors, hormones or non-steroidal anti-inflammatory agents should also be excluded. All the included healthy peoples should be of good health with no obvious abnormalities in routine physical examinations. The study protocol was authorized and all the procedures performed in this study involving human participants were in strict consistence with the ethical standards of ethics Committee at the xxx Hospital and with the 1964 Helsinki declaration and its later amendment. Well-written informed consent was obtained from all the participants prior to their enrollments.

### Sample procession and detection method

A volume of 100ul serum samples were thawed, deproteinized with the volume of 400ul acetonitrile, and centrifuged at 14000r / min for 5 minutes. Each sample was processed by Agilent 1200 high performance liquid chromatography combined with a 6520 accurate electrospray ionization /quadrupole-time-of-flight mass system (Agilent Technologies, California, USA). Serum samples were separated on an Eclipse Plus C18 column (2.1×150mm, 3.5μm, Agilent Technologies, USA), with the condition of 180 μl injection volume, 0.8 ml/min flow rate and 45°C column temperature, by using a gradient program of the mobile phase A 0.1% formic acid solution (ESI^+^)/water (ESI^-^) and mobile phase B was acetonitrile with 0.1% formic acid solution (ESI^+^)/ acetonitrile (Merck, Darmstadt, Germany) (ESI^-^). The gradient program started from 20% B for 0-1.5 min, linear increased from 20 to 95% B for 1.5-7 min, stayed at 95% B for 7-9.9min, and then linear decreased from 95 to 20% B for 9.9-10 min and equilibrated for 20% B for 10-11 min. To avoid cross-contamination from GC patients, all the serum samples of healthy people were injected at the end.

All the data were collected in ionization quadrupole-time-of-flight mass spectrometry with both positive (ESI^+^) and negative (ESI^-^) full scan mode find either basic or acidic biological compounds in human serum, which may be specific and sensitive biomarkers for GC. The conditions of mass spectrometry were as following: the capillary voltage 3.2kv; the cone voltage 35V; the desolvation temperature 350°C; the source temperature was 100°C; the desolvation gas (nitrogen) flow rate 650L/h; the cone gas (nitrogen) flow rate was 50L/h; a mass range of 50 to 1000; scan time of 1s and inter-scan delay of 0.02s.

### Data processing and statistical analysis

Firstly, both full-scan ESI^+^ and ESI^-^ raw mass spectra was gathered by using data-acquisition software Analyst TF 1.5.1 (AB Sciex, California, USA). Then, the data like retention time, peak area and m/z ratio, was generated by using Marker View 1.2 (AB Sciex, California, USA) and the related serum metabolites were structurally confirmed by comparing the m/z ratio and ion mode of those metabolites with data shown in HMDB (www.hmdb.ca) databases. Subsequently, principal component analysis (PCA) was adopted in score plots to make a distinction between the similarity or difference of the scatters between GC patients and healthy controls, and thereby two sample t-test by using SPSS 22.0 (SPSS Inc., Chicago, USA) was performed to select potential biological variables which statistically significant different between GC patients and healthy controls. Additionally, the hierarchical clustering analysis was implemented by using BRB-Array Tools (Dr. Richard Simo & BRB-Array Tools Development Team, USA) to discriminate the subgroups and partial least squares discriminant analysis (PLS-DA) was applied by using simca-p software (Umetric AB, CA, USA) to identify the statistical important serum metabolites between GC patients and healthy controls. VIP plots of PLS-DA were drawn to ensure the correct potential serum biomarkers for both GC patients and healthy controls. The intensity of the background interference was normalized by using the global median subtraction method.

All the 125 GC patients were clustered into serum metabolites selected from the PLS-DA analysis, and differences in clinicopathological parameters like tumor differentiation, age, vascular invasion, TNM staging, survival rate, levels of Ki-67 and P53 and tumor position and expression were observed among the subgroups by chi-square test or variance analysis. The Kaplan-Meier (K-M) survival curve of each group was plotted based on different thresholds of sensitivity and specificity of survival time in order to find the most potential serum biomarkers for GC. Finally, multivariate COX regression analysis, variance analysis and K-M survival curve were used to find the independent prognostic factor for GC in human serum. All the statistical significance was set to be *p*< 0.05.
